# Integrative Computational Approaches to Prostate Cancer with Conditional Reprogramming and AI-Driven Precision Medicine

**DOI:** 10.3390/cells15080700

**Published:** 2026-04-15

**Authors:** Ahmed Fadiel, Punit Malpani, Kenneth D. Eichenbaum, Frederick Naftolin, Aya Hassouneh, Geralyn Chong, Kunle Odunsi

**Affiliations:** 1 Computational Oncology Unit, The University of Chicago Comprehensive Cancer Center, Chicago, IL 60637, USA; afadiel01@uchicago.edu (A.F.); punitm@uchicago.edu (P.M.);; 2 Department of Anesthesiology, Oakland University William Beaumont School of Medicine, Royal Oak, MI 48309, USA; 3 Department of Anesthesiology, Wayne State University, Detroit, MI 48341, USA; 4 e-Bio Corporation, New York, NY 10001, USA; fnaftolin2@gmail.com; 5 Department of Electrical and Computer Engineering, Western Michigan University, 1903 W. Michigan Ave, Kalamazoo, MI 49008, USA; aia.hassouneh@gmail.com; 6 University of Chicago Medicine Comprehensive Cancer Center, 5481 South Maryland Avenue, MC1140, Chicago, IL 60637, USA; odunsia@uchicago.edu; 7 Department of Obstetrics and Gynecology, University of Chicago, Chicago, IL 60637, USA

**Keywords:** prostate cancer, conditional reprogramming, single-cell multi-omics, spatial transcriptomics, neuroendocrine transdifferentiation, physics-informed machine learning, digital twins, lineage plasticity, FOXA2–NKX2-1, precision oncology

## Abstract

Prostate cancer, particularly metastatic castration-resistant prostate cancer (mCRPC), presents therapeutic challenges rooted in adaptive lineage plasticity and neuroendocrine transdifferentiation. Conventional genome-based models fail to account for the divergent clinical trajectories observed among tumors that share identical driver mutations. This limitation requires reconceptualizing cancer as a dynamic system in which tumor cells can execute context-dependent molecular programs governed by epigenetic and transcriptional network remodeling. This review critically evaluates three convergent technological pillars reshaping prostate cancer research and clinical care. First, conditional reprogramming (CR) enables the rapid generation of patient-derived models that preserve genomic fidelity, intratumoral heterogeneity, and reversible phenotypic plasticity without genetic manipulation. Second, single-cell and spatial multi-omics approaches have clarified the cellular trajectories underlying luminal-to-neuroendocrine transdifferentiation, identifying a therapeutically actionable intermediate state. They have revealed the hierarchical transcription factor network (FOXA2–NKX2-1–p300/CBP) which orchestrates chromatin remodeling during this lethal transition. Third, physics-informed machine learning and digital twin architectures aim to move beyond correlative risk prediction toward mechanistically sound forecasting of tumor evolution, treatment response, and resistance emergence. We address unresolved challenges in prospective clinical validation, spatial heterogeneity capture, regulatory pathways for functional diagnostics, and the imperative for causal, as opposed to associative, inference from perturbational datasets. The integration of these three domains through closed-loop experimental–computational feedback cycles represents a paradigm shift from reactive to anticipatory precision oncology.

## 1. Introduction: Cancer as a Dynamic Adaptive System

Prostate cancer (PCa) remains the second most commonly diagnosed malignancy in men worldwide, with an estimated 299,010 new cases and 35,250 deaths projected in the United States for 2024 alone [1,2]. The clinical landscape has evolved considerably since the introduction of next-generation androgen receptor (AR) pathway inhibitors including enzalutamide, abiraterone, apalutamide, and darolutamide. However, a subset of patients inevitably progresses to metastatic castration-resistant prostate cancer (mCRPC). Among these, treatment-emergent neuroendocrine prostate cancer (t-NEPC) has emerged as a lethal adaptive phenotype in approximately 15–25% of advanced cases [3,4], representing one of the most clinically intractable manifestations of therapy-induced lineage plasticity in solid oncology.

What makes the mCRPC–NEPC transition so conceptually challenging is that it exposes the inadequacy of purely genomic explanatory frameworks. Tumors harboring identical TP53/RB1 co-loss, which is widely considered a key genetic prerequisite for lineage plasticity, follow wildly divergent clinical trajectories [5,6]. Some remain AR-dependent adenocarcinomas for years, while others undergo rapid neuroendocrine conversion within months of androgen receptor signaling inhibitor (ARSI) exposure. This clinical heterogeneity cannot be reconciled with a model in which mutations deterministically dictate phenotype. Instead, cancer cells function as adaptive systems executing context-dependent molecular programs. Such divergent phenotypic outcomes arise when cellular states are governed predominantly by epigenetic reprogramming, transcriptomic rewiring, and signaling network adaptation rather than by new genetic mutations [7,8].

**Figure 1:**
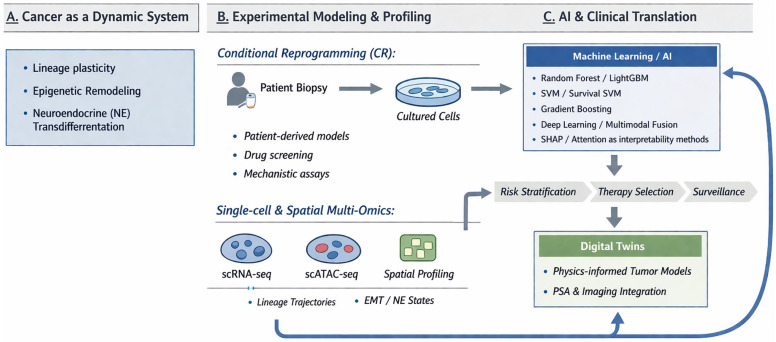
A dynamic systems framework for prostate cancer evolution integrating patient-derived models, single-cell profiling, and AI-enabled clinical translation. Schematic overview of an integrated pipeline that treats prostate cancer as a dynamic system (**A**), characterized by lineage plasticity, epigenetic remodeling and neuroendocrine (NE) transdifferentiation. Patient biopsies are subjected to conditional reprogramming (CR) to generate cultured, patient-derived models for drug screening and mechanistic assays, and for single-cell and spatial multi-omics profiling (scRNA-seq, scATAC-seq and spatial profiling) to define lineage trajectories and epithelial–mesenchymal transition (EMT)/NE states (**B**). These experimental data feed AI-based models (**C**)—including ensemble learning (Random Forest, LightGBM, gradient boosting), support vector machines (SVM/survival SVM), deep learning and multimodal fusion approaches, with interpretability frameworks such as SHAP and attention mechanisms—to support risk prediction and diagnosis, prognosis and recurrence assessment, and inference of evolutionary indicators. AI-driven outputs enable clinical decision support across risk stratification, therapy selection and surveillance, and are further integrated into physics-informed digital twin tumor models incorporating prostate-specific antigen (PSA) dynamics and imaging data, establishing a closed-loop framework that links biological modelling with AI-enabled clinical translation.

Viewing tumors not as static genetic entities, but as dynamic computational systems with potentially predictable evolutionary trajectories is directly relevant for developing a therapeutic strategy. If phenotypic plasticity is governed by reversible epigenetic states rather than irreversible genetic mutations, then the therapeutic objective shifts from killing resistant clones to controlling lineage fate decisions. Achieving this requires a new kind of integrative infrastructure: patient-derived experimental models that faithfully recapitulate native plasticity, multi-scale omics profiling that resolves the cellular logic of state transitions, and computational frameworks capable of predicting and pre-empting the evolutionary trajectories of individual tumors under therapeutic pressure. Figure 1 provides an overview of this dynamic systems framework, integrating patient-derived models, single-cell multi-omics, and AI-enabled clinical translation.

This review examines the convergence of three main pillars that are reshaping prostate cancer research and clinical care: conditional reprogramming, single-cell multi-omics, and artificial intelligence. Rather than merely cataloguing recent advances, this review emphasizes their integration through closed-loop feedback cycles, where computational predictions are continuously validated and refined by patient-derived experimental systems. Recent systematic evaluations of AI-driven digital twin technologies further reinforce this vision, moving from static risk assessment toward dynamic, individualized models capable of assimilating longitudinal clinical, imaging, and molecular data. This shift requires bridging significant unresolved challenges in real-time data fusion, model reliability, and prospective validation [9].

## 2. Conditional Reprogramming: Patient-Derived Models Preserving Tumor Heterogeneity

### 2.1. Principles and Molecular Mechanisms

Conditional reprogramming (CR) technology enables the indefinite propagation of primary epithelial cells without genetic manipulation through co-culture with irradiated 3T3-J2 feeder cells in the presence of the Rho-associated kinase (ROCK) inhibitor Y-27632 [10,11]. This deceptively simple two-component system induces a transient proliferative state through coordinate activation of β-catenin and MYC signaling coupled with suppression of p53-mediated senescence, permitting genomic stability across more than 20 passages in reported studies [12,13]. The mechanistic elegance of CR lies in its reversibility: upon ROCK inhibitor withdrawal or xenografting into immunodeficient mice, CR cells undergo differentiation with 10- to 100-fold upregulation of luminal markers (AR, NKX3.1, PSA) and concurrent suppression of basal progenitor markers (KRT5, KRT14, p63) [14,15]. This reversible plasticity suggests that CR preserves native cellular differentiation potential without inducing major permanent genetic alterations. This property distinguishes it from immortalized cell lines or iPSC-based approaches.

Whole-genome sequencing of CR cultures confirms preservation of patient-specific somatic alterations and copy-number variations that are lost or distorted in conventional cell line derivation [14]. This genomic fidelity, combined with the ability to establish cultures from biopsies as small as 5 mm^3^ within 7–10 days, positions CR as a tractable platform for functional precision oncology. This platform enables functional testing of therapeutic vulnerabilities informed by genomic profiling.

These mechanistic conclusions were supported by feeder-cell co-culture perturbation experiments, ROCK inhibitor withdrawal assays, xenograft differentiation studies, whole-genome sequencing, and lineage-marker profiling of epithelial and basal markers.

### 2.2. Applications in Prostate Cancer Research

CR technology has enabled the establishment of matched normal and tumor prostate epithelial cultures from individual patients. This advance is notable given the historical scarcity of primary prostate adenocarcinoma models [10,16]. The traditional workhorse cell lines of prostate cancer research—PC-3, DU145, and LNCaP—derive from metastatic sites and poorly represent the biology of primary disease. CR cultures, by contrast, preserve patient-specific mutational landscapes and intratumoral heterogeneity. Genomic analyses of CR models identify alterations in DNA repair pathways such as BRCA2, ATM, and PI3K–AKT–mTOR signaling, as well as chromatin remodeling complexes that reflect the diversity of primary tumor biology [14,16].

One interesting application of conditional reprogramming has been the modelling of neuroendocrine transdifferentiation. Ci et al. established CR cultures from the LTL331 patient-derived xenograft, which undergoes spontaneous adenocarcinoma-to-NEPC transition following castration [17]. The resulting LTL331-CR cells retained parental tumor mutations (TP53^R248W^, RB1^−/−^), permitted ex vivo genetic manipulation (including SOX2 knockdown to assess its necessity for the NE transition), and recapitulated NEPC histopathology upon murine engraftment. This system provided the first tractable platform for mechanistic interrogation of the luminal-to-NEPC transition outside the constraints of in vivo xenograft timelines.

Wang et al. subsequently demonstrated clinical integration potential by performing integrative multi-omics analysis consisting of whole-genome sequencing, phosphoproteomics, and metabolomics. Their CR cultures were subjected to high-throughput drug screening across panels of 50–100 compounds [16]. They identified patient-specific vulnerabilities, including PARP inhibitor sensitivity in BRCA2-wildtype tumors that nonetheless exhibited homologous recombination deficiency (HRD) signatures. These would have been missed by genomics-only approaches. This work illustrated that functional vulnerability often diverges from predictions by genomic profiling alone. The experimental interrogation enabled by CR cultures captures biological complexity that computational models cannot yet fully anticipate.

Table 1 compares major patient-derived model systems used in prostate cancer research, highlighting their relative strengths, limitations, and suitability for functional studies and drug screening.

## 3. Single-Cell and Spatial Multi-Omics: Decoding Lineage Plasticity and Cellular Decision Logic

### 3.1. Evidence Supporting Luminal Origin of Prostate Cancer

The cellular origin of neuroendocrine prostate cancer was, until recently, a matter of controversy. Competing hypotheses posited derivation from basal progenitors, neural crest remnants, or trans-differentiation of luminal adenocarcinoma cells. Single-cell RNA sequencing has provided strong evidence clarifying this debate. Dong et al. profiled 21,292 cells from needle biopsies of six mCRPC patients and demonstrated that neuroendocrine tumor cells exhibit a luminal-like epithelial transcriptional signature rather than basal or neural crest characteristics [19]. Lineage trajectory inference using RNA velocity and pseudo-time algorithms confirmed that focal neuroendocrine differentiation originates exclusively from AR-positive luminal malignant cells, not from basal compartments. This provides strong support for the luminal-to-neuroendocrine transdifferentiation model [19,20]. In addition, genes identified in mediating cell plasticity and cell fate transitions are under the strict control of elaborate regulatory networks formed by various noncoding RNAs [21]. This adds another layer of complexity, since the disruption of the noncoding RNA networks may, in fact, be more crucial to cancer pathogenesis than oncogene mutations.

The temporal dynamics of this transition have been mapped with granularity. Longitudinal scRNA-seq of the LTL331 PDX model across eight timepoints, from pre-castration through NEPC emergence, revealed a highly regulated sequence of cellular state transitions. High-AR adenocarcinoma cells are depleted following androgen deprivation, replaced by AR^low^ populations that transit through an AR^−^/NE^−^ intermediate state enriched for epithelial–mesenchymal transition (EMT). Neural crest stemness markers are expressed (SNAI2, SOX2, CD44) before committing to the NEPC lineage [22]. The existence of this AR^−^/NE^−^ intermediate, a state that—neither adenocarcinoma nor NEPC but poised for lineage commitment—may represent a therapeutically vulnerable window. Intercepting cells at this juncture, before epigenetic locks consolidate neuroendocrine identity, may be far more effective than attempting to reverse a fully established NEPC phenotype.

The robustness of neuroendocrine gene signatures has been scrutinized through integrative meta-analysis. Liu et al. analyzed 210,879 single cells from 66 tumor samples across 9 independent cohorts, applying machine-learning-based batch correction and marker stability scoring to identify 762 high-confidence neuroendocrine markers [23]. A notable finding was the poor cross-dataset concordance of previously published NE gene signatures, including canonical markers ENO2 and CHGA. This reinforces that the field’s biomarker toolkit requires rigorous recalibration against multi-cohort single-cell reference atlases before clinical deployment.

### 3.2. Chromatin Remodeling and Epigenetic Drivers of Lineage Plasticity

**Figure 2:**
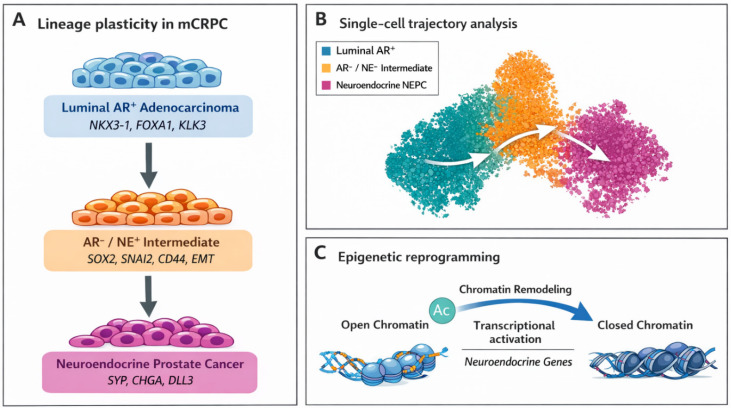
Single-cell evidence for luminal-to-neuroendocrine lineage plasticity in prostate cancer. (**A**) Conceptual model of lineage plasticity in metastatic castration-resistant prostate cancer (mCRPC). Androgen receptor-positive luminal adenocarcinoma cells undergo adaptive transcriptional reprogramming following androgen deprivation, transitioning through an AR^−^/NE^−^ intermediate state enriched for epithelial–mesenchymal transition (EMT) and stemness-associated programs before committing to the neuroendocrine prostate cancer (NEPC) lineage. (**B**) Schematic UMAP representation of single-cell transcriptomic profiles showing cellular states along the transition, including luminal AR^+^ cells (NKX3-1^+^, FOXA1^+^, KLK3^+^), transitional intermediate cells expressing EMT and stemness markers (SOX2^+^, SNAI2^+^, CD44^+^), and NEPC cells expressing neuroendocrine markers (SYP^+^, CHGA^+^). RNA velocity vectors indicate the inferred directionality of lineage transitions from luminal adenocarcinoma toward neuroendocrine states. (**C**) Epigenetic reprogramming associated with this transition involves chromatin accessibility changes and remodeling of transcriptional regulatory networks enabling activation of neuroendocrine gene programs. Adapted from Dong et al. [19], Liu et al. [23], and Lu et al. [26].

Computational modeling that treats cellular identity as a Waddington Landscape demonstrates how epigenetic blockade can regulate luminal prostate cells and their transition to neuroendocrine cells (Figure 2) [24]. Inhibitors of the enzyme EZH2 are being considered to prevent reshaping of DNA and the resulting gene silencing and neural-like gene activation [25]. The mechanism of neuroendocrine transdifferentiation has been examined by multi-omic integration of scRNA-seq with ATAC-seq, HiChIP, and DNA methylation profiling. Lu et al. identified distinct three-dimensional chromatin architectures distinguishing NEPC from castration-resistant adenocarcinoma, revealing that the transition involves wholesale reorganization of the genome’s spatial topology [26]. At the center of this reorganization sits a hierarchical transcription factor network where pioneer factor FOXA2 initiates binding at previously inaccessible neuroendocrine enhancers. This mediates regional DNA demethylation that makes these loci competent for transcription factor occupancy. This chromatin priming induces expression of the neural transcription factor NKX2-1, which preferentially occupies gene promoters and engages enhancer-bound FOXA2 via chromatin looping. This creates a feed-forward circuit. The FOXA2–NKX2-1 complex then recruits p300/CBP histone acetyltransferase complexes to catalyze H3K27 acetylation at neuroendocrine enhancers, activating transcriptional programs governing synaptic vesicle proteins (SYP), neural cell adhesion molecules (NCAM1), and delta-like ligand 3 (DLL3) [26].

Pharmacological inhibition of p300/CBP has emerged as a potential therapeutic strategy, where A-485 abrogates neuroendocrine gene expression and suppresses NEPC tumor growth in vivo [26]. This finding suggests that targeting this epigenetic machinery could represent a viable therapeutic strategy. In addition, it validates the conceptual framework that lineage transitions are governed by targetable enzymatic activities rather than irreversible genetic alterations. Single-cell multiome analyses of isogenic cells undergoing time-course neuroendocrine transformation further revealed individual cells caught in intermediate epigenetic and transcriptomic states. This direct visualization of the transition in progress confirms that the process is gradual rather than switch-like [26].

### 3.3. Spatial Resolution of the Tumor Microenvironment

A fundamental limitation of dissociated single-cell approaches is the loss of spatial context. These approaches disrupt the positional relationships between cell states, stromal niches, and vascular architecture that govern local signaling and drug penetration [27]. Emerging spatial multi-omics technologies are beginning to address this gap. Spatial transcriptomics platforms (10x Visium, Xenium In Situ) combined with multiplexed protein imaging (CODEX, MIBI-TOF) now enable simultaneous mapping of transcriptomic states and protein expression within intact tissue architecture [28,29]. In prostate cancer specifically, these approaches have begun to reveal the spatial organization of immune exclusion zones around neuroendocrine foci, the perivascular niches that harbor treatment-resistant stem-like cells, and the gradients of AR signaling activity that predict local response heterogeneity.

Kfoury et al. applied single-cell profiling to human prostate cancer bone metastases and uncovered an actionable immunosuppressive microenvironment characterized by M2-polarised macrophages and regulatory T cells concentrated in the peritumoral stroma [29]. The integration of such spatial immune landscapes with tumor cell-intrinsic lineage states can help explain why immunotherapy has underperformed in prostate cancer relative to other solid tumors and where spatial biomarker-guided therapeutic combinations might overcome this resistance.

Table 2 summarizes the key molecular features defining prostate cancer lineage states during neuroendocrine transdifferentiation, with emphasis on transcriptional markers, epigenetic features, and therapeutic vulnerabilities.

## 4. Machine Learning and Artificial Intelligence in Prostate Cancer

Representative AI approaches applied in prostate cancer include ensemble learning methods such as Random Forest, LightGBM, and gradient boosting; support vector machines (SVM/survival SVM); deep learning and multimodal fusion approaches; and interpretability frameworks such as SHAP and attention mechanisms.

### 4.1. Diagnostic Risk Prediction: Beyond PSA

Machine learning models integrating clinical parameters, multiparametric MRI (mpMRI) radiomics, and laboratory values have consistently demonstrated superiority over PSA-based screening for prostate cancer detection [32,33,34,35]. The magnitude of improvement is substantial. For example, a random forest classifier trained on clinical and laboratory features achieved an AUC of 0.918 for PCa diagnosis in a cohort of 551 patients [32]. Similarly, Light Gradient Boosting Machine (LightGBM) models trained on 5122 clinical records distinguished PCa from benign prostatic hyperplasia (BPH) with an AUC of 0.93 and sensitivity of 86% [35]. These approaches illustrate how ensemble learning methods can integrate heterogeneous clinical variables to improve diagnostic discrimination relative to PSA-derived metrics alone. In a prospective Korean cohort of 41,837 participants, survival support vector machines incorporating metabolic syndrome parameters achieved a C-index of 0.862 for incident prostate cancer (PCa) prediction over ten years [36]. This study demonstrates the potential of machine learning-based survival models to move beyond immediate diagnostic classification toward long-term population-level risk prediction, enabling identification of individuals at elevated future risk before clinical detection.

What these performance metrics obscure, however, is a deeper inherent limitation. Current diagnostic ML models are correlative in that they identify statistical patterns between input features and biopsy outcomes without encoding biological mechanisms. A random forest does not ‘understand’ that PSA arises from kallikrein gene expression in luminal epithelium. It merely learns that certain PSA trajectories correlate with certain histological outcomes. This distinction matters because purely correlative models often lack robustness when applied across different clinical populations. Their performance degrades when deployed in populations whose feature distributions differ from training data, a problem that has afflicted nearly every ML model transported across institutional and demographic boundaries in oncology [13,37]. The path forward requires not only larger training datasets but architecturally different models that incorporate biological priors. This includes the physics-informed approaches discussed in Section 4.3.

### 4.2. Prognosis and Treatment Response Prediction

Prognostic machine learning has demonstrated robust performance in predicting biochemical recurrence (BCR) and therapeutic resistance. Weighted gene co-expression network analysis (WGCNA) identified 16 BCR-associated genes that form a prognostic signature with a hazard ratio of 3.2 (95% CI 2.1–4.9) in multivariate analysis [38]. Deep learning architectures integrating mpMRI radiomics with clinical–pathological features (iBCR-Net) achieved a 12.8-fold improvement in BCR prediction accuracy versus the CAPRA score, with decision curve analysis confirming clinical net benefit [39]. For metastatic disease, gradient boosting machines predict lymph node metastasis in intermediate- and high-risk patients with F1-score 0.838 and AUC 0.804 in multi-institutional validation [40].

Recent advances in machine learning have enabled integration of single-cell-informed evolutionary predictors narrowing the gap between biological resolution and clinical applicability. Models trained on longitudinal scRNA-seq profiles can quantify subclone expansion dynamics under therapeutic selection, potentially forecasting resistance to androgen receptor (AR) antagonists and immunotherapies before clinical evidence of progression [31]. Early implementations demonstrate that transcriptional programs associated with AR pathway reactivation and lineage plasticity can be detected at single-cell resolution prior to clinical progression, enabling predictive models to infer emerging resistant subclones during therapy. Complementary machine learning approaches have also been applied to predict drug chemosensitivity from genomic and transcriptomic tumor profiles, where supervised models trained on pharmacogenomic datasets estimate patient-specific response probabilities to therapeutic agents. For example, supervised learning models trained on large pharmacogenomic datasets can infer sensitivity to androgen receptor pathway inhibitors and cytotoxic agents from tumor molecular profiles, demonstrating the feasibility of algorithmically guided treatment selection. Such models illustrate the broader potential of ML to guide treatment selection by anticipating therapeutic resistance and identifying drugs with the highest predicted efficacy for individual tumors. This approach, using a tumor’s own evolutionary trajectory, resolved at single-cell resolution and input to predictive algorithms, represents a qualitative leap beyond static genomic risk scores. It treats the tumor as more than a fixed entity. Tumors represent a dynamic population with evolutionary behavior that can, in principle, be anticipated. With equal emphasis on machine learning and its merits in dynamic tumor modeling, clinicians must also consider the retrospective data often required in training statistical models that may encapsulate biases in institutional practices requiring strong prospective validation for clinical application.

### 4.3. Digital Twin Models for Predicting Patient-Specific Disease Trajectories

An exciting paradigm in computational oncology integrates mechanistic tumor growth models with machine learning to create patient-specific digital twins. Camacho-Gómez et al. developed a physics-based framework that reconstructs prostate tumor growth from serial PSA measurements by coupling MRI based digital anatomies with cellular parameters. These include proliferation rate, local vascularization, and oxygen transport within a reaction-diffusion model of tumor expansion linked to PSA production kinetics [41]. Following calibration from a baseline MRI and PSA measurement, the digital twin predicts over 900 days of tumor evolution from subsequent PSA measurements alone. Computation times are approximately 23 s per patient. This approach could reduce the frequency of surveillance MRI while maintaining monitoring accuracy.

What distinguishes physics-based approaches from purely data-driven ML is the incorporation of biological constraints as inductive biases. A reaction-diffusion model ‘knows’ that tumor growth is bounded by oxygen diffusion gradients, and that PSA secretion scales with viable tumor cell mass. These constraints restrict the model’s hypothesis domain to physically plausible solutions. This improves generalization from limited patient data and provides mechanistically interpretable predictions. This is also in direct contrast to the ‘black box’ outputs of conventional deep learning. Emerging frameworks incorporating SHAP (SHapley Additive exPlanations) values, attention mechanisms, and Bayesian uncertainty quantification further enhance interpretability while maintaining predictive performance [42]. The integration of Gompertzian growth ordinary differential equations with neural network components creates hybrid architectures where known biology constrains what the model can learn. Neural network components capture residual complexity that mechanistic models alone cannot represent.

A complementary framework of digital twin models was demonstrated by John et al. (2026) [43]. The study applied doubly robust estimators (AIPW/DR) to a cohort from the MIMIC-IV dataset for estimating counterfactual outcomes of various drug treatments. These treatments spanned various clinical endpoints such as mortality, ICU transfers, emergency department repeat visits, and length of stay. These causal estimates of average treatment effects were then used to construct policies mapping individual patient characteristics to treatment recommendations that not only maximize potential, but also minimize patient harm. This study highlights the benefit of digital twins. They can rely on more than mechanistic tumor simulation to produce reliable and actionable patient-specific prediction. Both studies highlight an exciting application of digital twins in simulating treatment intervention and patient monitoring for increased response rates, drug awareness, and overall patient safety.

Table 3 provides a concise overview of representative machine learning applications in prostate cancer, including their model architectures, performance, validation setting, and interpretability methods.

Anticipatory oncology models can use ML for structured data integration and risk stratification for recurrence prediction. This is done using algorithms such as Gradient Boosting, Support Vector Machines or Random Forrest to identify shifts in receptor activity. ML can also help with feature extraction in CT, PET, or MRI imaging that are difficult for the human eye to identify [46]. ML ensembles can also stack various models to resolve multi-omic changes [47]. These models can also leverage the neural networks of Deep Learning to resolve unstructured data and complex patterns inaccessible with manual feature extraction. One example is the use of Shapley additive explanations [48]. Convolutional neural networks can analyze pathology images to learn and flag cellular atypia [49]. Temporal anticipation is moving toward longitudinal data analysis using Long Short-Term Memory (LSTM) and Transformer techniques which can identify the trend changes in biomarkers like ctDNA [50]. Combined with multi-modal fusion, DL can integrate genomic and proteomic data with electronic health records and environmental data from wearables [51].

## 5. Integration Toward Anticipatory Precision Oncology

### 5.1. The Closed-Loop Experimental–Computational Framework

The three pillars reviewed here include conditional reprogramming, single-cell multi-omics, and machine learning. All achieve their maximal impact through systematic integration within a closed-loop framework. Conditional reprogramming provides renewable, patient-specific biological material for experimental perturbation and drug screening. When CR cultures are subjected to single-cell multi-omics profiling during drug exposure or lineage transition, they generate the high-dimensional trajectory data that machine learning models require for training. Computational models trained on these trajectories make predictions about future tumor behavior. These predictions can be experimentally tested in the same CR system, closing the loop.

**Figure 3:**
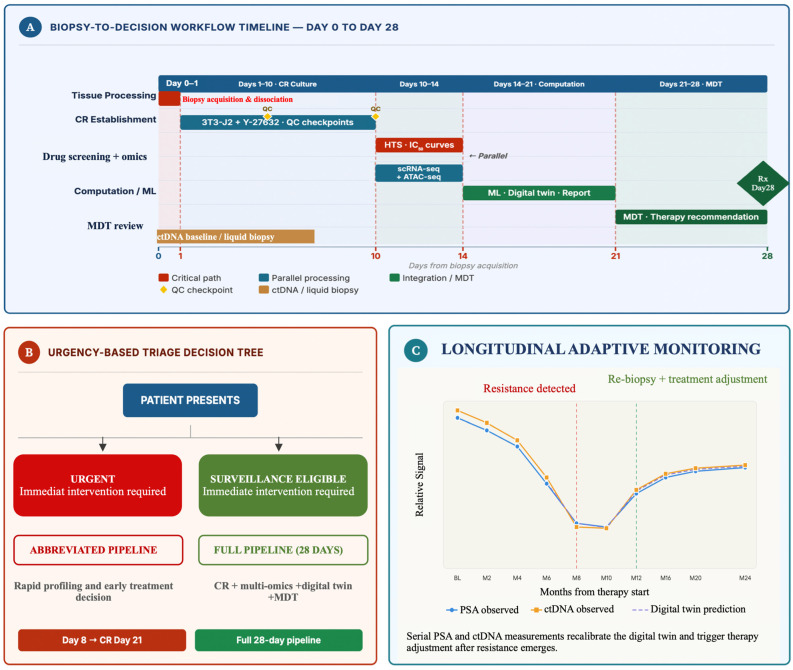
Clinical implementation roadmap and timeline for integrated CR–multi-omics–AI precision oncology in prostate cancer. (**A**) Gantt-style workflow timeline from biopsy to therapeutic decision: Day 0–1 (biopsy acquisition and tissue processing), Days 1–10 (CR culture establishment with real-time quality checkpoints), Days 10–14 (parallel high-throughput drug screening + scRNA-seq/ATAC-seq profiling), Days 14–21 (computational integration, ML model inference, and digital twin calibration), Day 21–28 (multidisciplinary tumor board integration and therapy recommendation). Critical path elements in red; parallel processing in blue. (**B**) Decision tree for triage-based clinical deployment: patients stratified by urgency (immediate intervention required vs. surveillance-eligible) with corresponding abbreviated vs. full pipeline deployment. (**C**) Longitudinal monitoring schema showing integration of serial ctDNA measurements with digital twin predictions and periodic CR-based functional re-evaluation, enabling dynamic therapy adjustment. Arrows indicate data flow between real-world clinical monitoring and in-silico predictions.

Proof-of-concept integration strategies have demonstrated clinical feasibility. Computational models trained on scRNA-seq trajectories from the LTL331 PDX system successfully predicted the temporal window for EZH2 inhibition to block NEPC emergence [22]. This prediction was subsequently validated experimentally. Multimodal fusion significantly enhances predictive robustness. In hormone-sensitive prostate cancer, models combining proteomics, mpMRI, and histopathology improved progression prediction (ΔC-index +0.14) over single modal approaches [44]. Radiomic–immunologic fusion models achieved 92% accuracy in discriminating progression states by integrating spatial protein expression with MRI texture features [45]. These multimodal gains reflect the complementary information content. Genomics captures mutational architecture, transcriptomics captures current gene expression programs, radiomics captures macroscopic tissue phenotypes, and spatial omics captures the ecological context in which cells make fate decisions. Figure 3 summarizes this proposed closed-loop workflow, from biopsy acquisition and model generation to clinical decision-making and longitudinal monitoring.

### 5.2. Clinical Translation: Barriers and Pathways

Despite promising preclinical validation, barriers impede clinical implementation of integrated computational–experimental platforms. These barriers include limited validation across diverse cohorts, delayed processing times, spatial sampling bias, regulatory and reimbursement challenges, and barriers to clinical adoption. These barriers are now discussed below.

#### 5.2.1. Validation and Generalizability

Most ML models in prostate cancer derive from single-institution cohorts with limited demographic diversity [13,37]. Performance in training cohorts routinely fails to translate across ethnic groups and healthcare systems. This concern is compounded by the well-documented underrepresentation of African American and Hispanic patients in genomic reference datasets. External validation in prospective, multi-center, demographically representative trials remain scarce.

#### 5.2.2. Temporal Constraints

With CR culture establishment requiring 7–10 days and comprehensive multi-omics profiling adding 14–21 days, urgent therapy initiation is a challenge. For example, in cases of symptomatic mCRPC with visceral crisis such timelines become prohibitive [15]. Accelerated molecular profiling pipelines using same-day drug sensitivity assays and rapid single-cell workflows are under development but are not yet clinically validated.

#### 5.2.3. Spatial Sampling Bias

A single needle biopsy captures less than 5% of total tumor volume, potentially missing resistant subclones or microenvironmental niches that drive adaptation [52]. Multi-region sampling strategies, while informative in research settings, remain logistically challenging in routine clinical practice. Liquid biopsy integration, combining CR-based functional testing of biopsy material with serial circulating tumor DNA (ctDNA) monitoring, offers a promising approach to capture temporal dynamics without repeated tissue sampling [30].

#### 5.2.4. Regulatory and Reimbursement Pathways

CR-based functional diagnostics currently lack established FDA clearance pathways. The closest regulatory precedent is the companion diagnostic model for targeted therapies, but CR-guided drug selection is fundamentally different because it tests a patient’s cells against a panel of agents rather than detecting a specific molecular target. Demonstrating clinical utility sufficient for reimbursement will require prospective randomized trials comparing CR-guided versus standard therapy selection. This is being conducted in trials such as NCT04879017 that are only now beginning [53,54].

#### 5.2.5. Clinical Adoption and Trust

Deep learning ‘black boxes’ fundamentally impede adoption in clinical oncology, where therapeutic decisions carry life-and-death consequences and clinicians demand mechanistic rationale. Physics-based architectures partially address this by encoding known biology as model constraints. They may help achieve the level of interpretability required for routine clinical use, allowing the treating oncologist to understand why the model recommends abiraterone over docetaxel for a specific patient. This remains an active area of development [42,55].

### 5.3. Future Research Directions: From Correlation to Causation

The most consequential intellectual challenge facing the field is the transition from correlative to causal inference. Nearly all current ML applications in prostate cancer identify associations. These include radiomics features that correlate with BCR, gene expression signatures that correlate with neuroendocrine score, and PSA trajectories that correlate with tumor growth. Correlation, however, does not identify intervention points. A gene whose expression correlates with resistance may be a bystander rather than a driver. Suppressing it may have no therapeutic effect. Causal inference frameworks, such as do-calculus, structural equation models, and perturbational machine learning applied to CRISPR/drug screens, offer a principled path from ‘what predicts outcome’ to ‘what, if perturbed, changes outcome’ [37,56]. This distinction is the difference between a biomarker and a therapeutic target.

Systematic evaluations indicate that next-generation digital twins must integrate mechanistic tumor models with machine learning capable of assimilating longitudinal multimodal data [9]. Hybrid architectures combining biological constraints, such as evolutionary game theory and reaction-diffusion dynamics, with real-time data updating and explainable prediction will require experimentally grounded platforms. In these platforms, iterative CR modelling coupled with multi-omics provides a training basis for computational refinement [9]. Research avenues with the greatest transformative potential include spatial multi-omics ecosystem mapping, federated models, and AI guided randomized trials.

Spatial multi-omics ecosystem mapping can help drive improved modeling. Combining spatial Visium and Xenium transcriptomics toolkits with multiplexed protein imaging such as CODEX and MIBI-TOF enables upgraded mapping capabilities for cellular ecosystems. These ecosystems include immune infiltrates, fibroblast niches, and vascular architecture, which govern local resistance and lineage transition [28,29]. This mapping can integrate evolutionary ecological modelling. This involves integration of ecological principles such as competitive release, niche construction, and frequency-dependent selection. Once integrated, these computational frameworks improve predictions of tumor adaptation under therapeutic pressure [57]. Adaptive therapy strategies can be designed to modulate treatment intensity to maintain therapy-sensitive populations. Such adaptive strategies may competitively suppress resistant clones, representing a direct clinical application of this ecological thinking.

Federated models enable multi-institutional training datasets that can include demographically diverse patient groups. These models can be designed to preserve patient privacy through encrypted gradient sharing and differential privacy mechanisms [58]. Federated methods can become standard practice for building ML models that generalize across populations. Validation of these platforms will transition over time from retrospective to prospective.

Rigorous randomized trials (e.g., NCT00268476) comparing AI-guided therapy selection versus standard care can help establish clinical utility and reimbursement pathways [59]. These trials can also be designed to adapt in real time. This would involve combining CR-based functional testing with serial ctDNA monitoring to dynamically adjust therapeutic strategies as resistance emerges. These new AI-guided randomized control trials would aid in closing the loop between molecular surveillance and therapeutic intervention [30].

## 6. Conclusions

The convergence of conditional reprogramming, single-cell multi-omics, and physics-based artificial intelligence establishes a qualitatively new framework for understanding and treating prostate cancer. CR technology provides renewable patient-specific models that preserve tumor heterogeneity and the functional plasticity that defines therapeutic vulnerability. Single-cell approaches have mapped the luminal-to-neuroendocrine transdifferentiation trajectory. They identified epigenetic master regulators in the FOXA2–NKX2-1–p300/CBP axis as druggable targets. Machine learning frameworks demonstrate clinical utility in risk stratification, progression prediction, and treatment selection. They integrate multimodal data within physics-based architectures that encode biological mechanism as inductive bias.

Prospective validation of integrated platforms is still in its infancy. Regulatory pathways for CR-based functional diagnostics are still largely undefined. The cost-effectiveness and accessibility of multi-omics profiling require systematic evaluation, particularly in the context of spatial and temporal tumor heterogeneity. Over time, experimental-computational integration can develop sophistication that current platforms only approximate.

Ultimately, prostate cancer is reconceptualized not as a static genomic entity amenable to one-time molecular classification. When viewed as a dynamic adaptive system, the evolutionary trajectory of prostate cancer may be predicted, intercepted, and potentially redirected through continuous experimental–computational feedback. The technologies reviewed here make this vision increasingly technically feasible. The remaining challenge is to build the clinical infrastructure, regulatory frameworks, and collaborative networks needed to translate this vision from proof of concept to standard of care.

## Figures and Tables

**Table 1:** Comparative Attributes of Patient-Derived Model Systems for Prostate Cancer Research.

Attribute	CR Cultures	Organoids	PDX Models	Traditional Cell Lines	Spheroids
Establishment time	7–10 days	2–4 weeks	3–6 months	Weeks–months	3–7 days
Success rate	70–90%	15–40%	10–30%	<5% primary	40–60%
Genomic fidelity	High (>20 passages)	High (early passages)	High (early passages)	Low (genetic drift)	Moderate
Tumor heterogeneity	Preserved	Partial	Preserved in vivo	Lost	Limited
Microenvironment	Absent	Partial (Matrigel)	Complete (murine)	Absent	Partial
Scalability for drug screening	High (2D expansion)	Moderate	Low (cost/time)	High	Moderate
Reversible plasticity	Yes (ROCKi-dependent)	Limited	Context-dependent	No	No
Regulatory readiness	Emerging	Emerging	Established (preclinical)	Established	Research only
Cost per model	Low–moderate	Moderate	High	Low	Low
Key limitations	No stroma/immune	Matrigel variability	Species mismatch	Non-representative	No architecture

CR, conditional reprogramming; PDX, patient-derived xenograft; ROCKi, ROCK inhibitor. Data synthesized from references [10,11,12,13,14,15,16,17,18].

**Table 2:** Molecular Markers Defining Prostate Cancer Lineage States During Neuroendocrine Transdifferentiation.

Lineage State	Key Transcription Factors	Surface/Cytoplasmic Markers	Epigenetic Features	Therapeutic Vulnerability
Luminal AR^+^ adenocarcinoma	AR, FOXA1, NKX3.1, HOXB13	KLK3 (PSA), TMPRSS2, KRT8, KRT18	AR-bound enhancers active; H3K27ac at luminal genes	ARSI (enzalutamide, abiraterone)
Transitional intermediate (AR^−^/NE^−^)	SOX2, SNAI2, ZEB1	CD44, ALDH1A1, VIM	Bivalent chromatin; EMT enhancers opening	EZH2 inhibitors; BET inhibitors
Early NEPC (focal)	FOXA2, ASCL1, NKX2-1	SYP, CHGA (low), DLL3	NE enhancers activating; FOXA2 demethylation	p300/CBP inhibitors (A-485); DLL3-targeting (rovalpituzumab)
Established NEPC (small cell)	NKX2-1, INSM1, NEUROD1	SYP, CHGA (high), NCAM1, ENO2	Locked NE chromatin; 3D reorganization complete	Platinum-based chemotherapy; Aurora kinase inhibitors
AR-indifferent (double-negative)	Noncanonical; MAPK-driven	AR^−^/NE^−^; variable surface phenotype	Distinct from NE; MAPK enhancer activation	MEK/ERK inhibitors; combination approaches

AR, androgen receptor; ARSI, androgen receptor signaling inhibitor; NE, neuroendocrine; NEPC, neuroendocrine prostate cancer; EMT, epithelial–mesenchymal transition. Compiled from references [19,20,22,23,26,30,31].

**Table 3:** Representative Machine Learning Applications in Prostate Cancer: Performance, Validation, and Limitations.

Application	Model Architecture	Performance	Dataset	Validation	Explainability	Reference
PCa diagnosis	Random Forest	AUC = 0.918	551 patients	Internal (70/30 split)	Feature importance	[32]
PCa risk (10-year)	Survival SVM	C-index = 0.862	41,837 participants	Prospective cohort	Cox regression comparison	[36]
Lymph node metastasis	Gradient Boosting	F1 = 0.838; AUC = 0.804	Multi-institutional	External validation	SHAP values	[40]
BCR prediction	Deep Learning (iBCR-Net)	12.8× vs CAPRA	Single institution	Internal	Decision curve analysis	[39]
PCa diagnosis (BPH)	LightGBM	AUC = 0.93; Sens = 86%	5122 records	Retrospective	Feature ranking	[35]
Progression prediction	Multimodal fusion	ΔC-index +0.14	Multi-omics	Internal + external	Attention mechanisms	[44]
Radiomic–immunologic	Fusion classifier	92% accuracy	Spatial + MRI	Internal	Heatmap overlays	[45]
Digital twin (PSA → tumor)	PINN + reaction-diffusion	>900-day prediction	Patient-specific MRI	4-patient validation	Mechanistic	[41]

AUC, area under the curve; BCR, biochemical recurrence; BPH, benign prostatic hyperplasia; CAPRA, Cancer of the Prostate Risk Assessment; PINN, physics-informed neural network; SHAP, SHapley Additive exPlanations; SVM, support vector machine.

## Data Availability

No new data were created or analyzed in this study.
